# How lipid ingestion is sensed: the mechanisms underlying intestinal hormone secretion

**DOI:** 10.1042/CS20250793

**Published:** 2026-09-10

**Authors:** Marta Santos-Hernández, Fiona M. Gribble, Frank Reimann

**Affiliations:** Institute of Metabolic Science Metabolic Research Laboratories, Addenbrooke’s Hospital, Cambridge, U.K.

**Keywords:** Enteroendocrine hormones, G-protein-coupled receptors, Lipid sensing

## Abstract

Dietary lipids are potent stimulators of intestinal hormone secretion, which plays a central role in the regulation of digestion, appetite, glucose homeostasis, and lipid metabolism. Following digestion in the intestinal lumen, lipid-derived molecules are detected through complementary mechanisms involving lipid transporters, nutrient-sensing receptors, intracellular metabolic pathways, and bile acid signaling. These pathways couple nutrient availability to the secretion of enteroendocrine hormones, including glucose-dependent insulinotropic polypeptide (GIP), glucagon-like peptide-1 (GLP-1), peptide YY (PYY), cholecystokinin (CCK), ghrelin and serotonin, thereby coordinating local and systemic metabolic responses. In the small intestine, CD36 contributes to lipid uptake and sensing, while intracellular lipid processing and chylomicron formation influence hormone secretion. Bile acids facilitate lipid digestion and absorption and further regulate enteroendocrine function through receptor-mediated signaling pathways. This review examines the cellular and molecular mechanisms underlying intestinal lipid sensing, with particular emphasis on the roles of CD36, chylomicron formation, bile acid signaling, and nutrient-sensing receptors in the regulation of enteroendocrine hormone secretion.

## Introduction

Lipids derived from the diet serve not only as essential energy nutrients but also as important signaling molecules that regulate a wide range of biological processes, including metabolism, appetite control, and hormone secretion [1]. In the gastrointestinal epithelium, fatty acids and other lipid species are detected by specialized enteroendocrine cells (EECs), primarily through G protein–coupled receptors (GPCRs). Activation of these receptors triggers the secretion of different peptide hormones that play key roles in coordinating intestinal function and systemic metabolic responses [2]. For instance, in response to luminal lipids, EECs release hormones such as cholecystokinin (CCK), glucagon-like peptide-1 (GLP-1), glucose-dependent insulinotropic polypeptide (GIP), ghrelin and serotonin (5-hydroxytryptamine, 5-HT), each exerting distinct physiological effects. CCK promotes gallbladder contraction and pancreatic enzyme secretion, thereby facilitating further lipid digestion, while also promoting satiety and slowing gastric emptying [3,4]. GLP-1 enhances glucose-dependent insulin secretion, inhibits glucagon release, delays gastric emptying and contributes to appetite suppression [5,6]. Similarly, GIP functions as an incretin hormone that enhances insulin secretion in response to nutrient intake, particularly of lipids and carbohydrates, and reduces further food intake whilst opposing nausea in mice [5,7]. Serotonin, produced by enterochromaffin cells (EC), regulates gut motility, secretion and visceral sensitivity, and also contributes to gut–brain signaling, promoting satiation and nausea [8]. By contrast, the hormone ghrelin is commonly referred to as the “hunger hormone” since it stimulates appetite and promotes food intake, and its secretion is generally suppressed following lipid ingestion [9]. Importantly, the magnitude and profile of gut hormonal changes depend on the type of fatty acid, as variations in chain length, degree of saturation and molecular structure can influence receptor activation and downstream signaling pathways.

In recent years, significant progress has been made in understanding the different molecular mechanisms underlying lipid sensing and the subsequent induction of gut hormone release. Emerging evidence highlights the importance of lipid absorption in the small intestine as a critical step in this process since some fatty acid–sensing receptors (FFARs) are localized on the basolateral membrane of EECs, suggesting that intracellular processing and transport of lipids are required for effective receptor activation [10]. Consequently, proteins and enzymes involved in lipid digestion, uptake and intracellular trafficking are essential modulators of hormone secretion.

This review aims to summarize current knowledge on the mechanisms of lipid sensing by EECs, with particular emphasis on the role of small intestinal lipid absorption and its impact on GPCR-mediated hormone release.

## Gut physiology

The gastrointestinal tract is composed of the small and large intestines, with the small intestine serving as the primary site of nutrient digestion and absorption. Within this system, epithelial stem cells located in intestinal crypts differentiate into multiple cell types, such as enterocytes, mucus-secreting goblet cells, defensin-secreting Paneth cells, which also support the local stem cell niche, tuft cells implicated in responses to helminth infections, and EECs. EECs account for approximately 1% of the total gut epithelial cell population and are dispersed throughout the intestines. Despite their low abundance, EECs secrete more than 20 hormones involved in digestion, intestinal motility, gastric emptying, the ileal brake, glucose homeostasis, and appetite regulation, making the gut the largest endocrine organ in the body [11]. Most EECs are classified as “open-type” cells, characterized by an apical surface with microvilli that directly contact the intestinal lumen. This structure enables them to detect physical and chemical stimuli. In response, they release a wide range of hormones that regulate gastrointestinal function, metabolism and food intake. Open-type EECs include L cells, which secrete GLP-1 and peptide YY (PYY); K cells, which produce GIP; and I cells, which release CCK. Some other EECs, particularly those in the stomach, are considered “closed-type” since they do not directly contact the lumen, although they still contribute to hormonal signaling. Examples include gastric A-like (X/A-like) cells, which secrete ghrelin, and gastric D cells, which release somatostatin (SST) [2].

The traditional classification of EECs has been based on morphology and the predominant hormone(s) they secrete in response to nutrient stimulation. Accordingly, L cells, K cells, and I cells are defined by their primary secretion of GLP-1, GIP, and CCK, respectively. However, accumulating evidence indicates that EECs display considerable heterogeneity in hormone expression, secretory profiles, and spatial distribution along the gastrointestinal tract [12,13]. This complexity has been highlighted by single-cell RNA sequencing analyses and immunohistochemical studies in mice [14,15]. Indeed, transcriptomic profiles of EECs from NeuroD1-Cre × Rosa26-EYFP reporter mice demonstrated co-expression patterns within the same EEC cluster, with *Gcg*-expressing cells in the small intestine also expressing *Cck*, while colonic L cells co-expressed *Gcg*, *Pyy*, and insulin-like peptide 5 (*Insl5*) [16,17]. In line with these observations, additional studies in mouse intestine [18–20], as well as single-cell RNA sequencing of chromogranin A (CHGA)-Venus–labeled human intestinal organoids [21], have identified multiple EEC populations characterized by combinatorial hormone expression. These observations suggest that although the classical classification remains useful, it should be interpreted as reflecting the predominant, but not exclusive, hormone produced by each cell type.

Despite the coexistence of multiple hormones within individual EECs, their secretory responses *in vivo* are stimulus-specific. Different studies indicate that these functional differences are influenced by factors such as the position of the cell along the gastrointestinal tract and its stage of maturation [12,13]. Variations in luminal pH, nutrient absorption, and the distribution of nutrient-sensing receptors along the gut determine the spatial organization of hormone release. Thus, the circulating gut hormone profile at any given time reflects both the composition and timing of recent nutrient intake, providing a dynamic and integrated readout of dietary signals entering the bloodstream.

Postprandial gut hormone secretion is primarily driven by the three major macronutrients—carbohydrates, fats and proteins. Stimulation of EECs typically occurs following nutrient digestion and, in many cases, subsequent absorption. These cells function as nutrient sensors through a broad array of membrane transporters and GPCRs. Activation of these receptors initiates intracellular signaling cascades, including increases in intracellular Ca^2+^ and cyclic adenosine monophosphate (cAMP), ultimately leading to hormone secretion [11,22]. Characterization of the GPCRs present across EEC subtypes, reported by quantitative PCR and RNA sequencing, revealed diverse and subtype-specific repertoires of nutrient-sensing receptors [21,23,24]. Of particular relevance to this review, lipid sensing represents a major regulatory mechanism governing intestinal hormone secretion and is a key pathway controlling intestinal hormone release and shaping postprandial endocrine responses. Several GPCRs have been implicated in lipid–dependent EEC activation, including long-chain and short-chain fatty acid receptors (FFAR1-4), the bile acid receptor GPBAR1 (also known as TGR5), the monoacylglycerol receptor GPR119, and the medium-chain fatty acid receptor GPR84. In addition, other receptor families, such as the olfactory receptors OR51E1 and OR51E2 and members of the ILDR family, have also been proposed to contribute to lipid sensing in EECs.

## Lipid sensing in the small intestine

**Figure 1:**
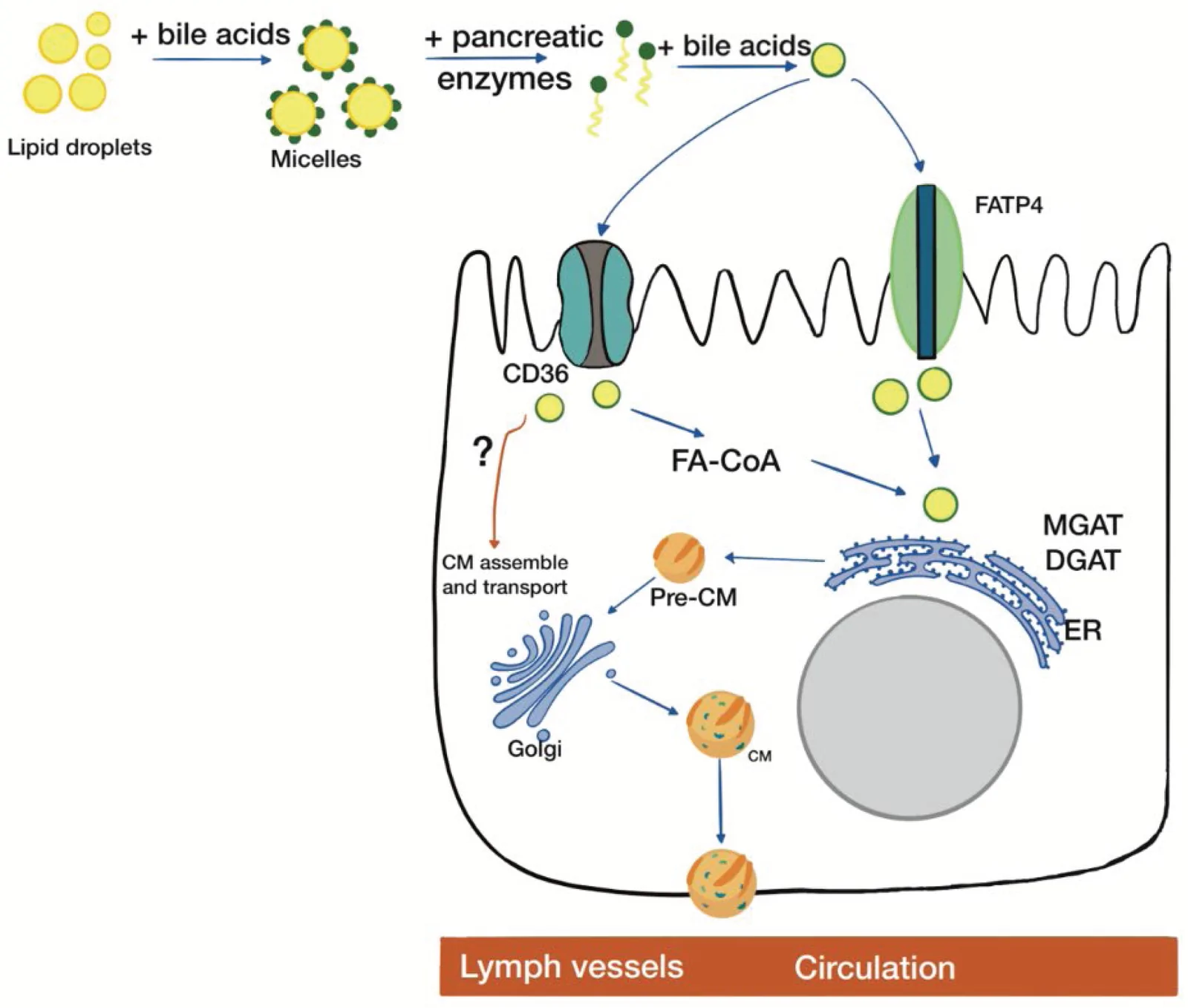
Schematic representation of intestinal lipid digestion and absorption Intestinal lipid digestion is primarily mediated by pancreatic lipases. FATP4 and CD36 facilitate lipid uptake into enterocytes and transport to the endoplasmic reticulum (ER). Within the ER, MGAT, and DGAT catalyze the sequential conversion of monoacylglycerol to diacylglycerol and then to triacylglycerol, respectively. The resulting lipids are incorporated into pre-chylomicrons (pre-CMs), which are transported to the Golgi apparatus for maturation into chylomicrons (CM) before being secreted. Abbreviations: fatty acid transport protein 4 (FATP4), fatty acid-binding protein 5 (FABP5), cluster of differentiation 36 (CD36), endoplasmic reticulum (ER), monoacylglycerol acyltransferase (MGAT), diacylglycerol acyltransferase (DGAT), pre-chylomicron (pre-CM), chylomicron (CM).

Dietary fat consists primarily of triacylglycerols (TGs), which provide approximately 90%–95% of the total energy derived from fat in the diet. The intestinal tract is the primary site of dietary fat digestion and absorption. After ingestion, TGs are emulsified by mechanical action in the stomach and bile acids in the proximal small intestine into small lipid droplets and micelles. These droplets are hydrolyzed by gastric and, more importantly, pancreatic lipases into free fatty acids and monoacylglycerols. Short- and medium-chain fatty acids (SCFAs, <C6, and MCFAs, C6–C10) readily diffuse into the enterocytes and are transported directly into the portal circulation bound to albumin [25,26]. By contrast, “free” long-chain fatty acids (LCFAs, >C12) are incorporated into mixed micelles formed by bile acids, which facilitate their delivery to the brush border of enterocytes. Uptake occurs via protein-mediated transport involving membrane-associated proteins such as CD36, FATP4 and fatty acid-binding proteins (e.g. liver and intestinal isoforms FABP1 and FABP2 in enterocytes, although K cells express high levels of FABP5) [27,28]. Once at the enterocyte surface, free fatty acids and monoglycerides dissociate from the micelles and are absorbed across the membrane, leaving the bile acids behind in the intestinal lumen. Inside the enterocyte, LCFAs and MAGs are transported to the endoplasmic reticulum, where they are re-esterified into TG and packaged into chylomicrons (Figure 1). These chylomicrons are subsequently processed in the Golgi apparatus, secreted into the lymphatic system, and ultimately delivered to peripheral tissues [29].

### Importance of CD36

CD36 is expressed on the apical surface of enterocytes in the duodenum and jejunum, where it facilitates the uptake of LCFAs [30]. Lipids in the intestinal lumen stimulate CD36-dependent up-regulation of proteins involved in chylomicron assembly, including microsomal triglyceride transfer protein and apolipoprotein B (apoB) [31]. Moreover, CD36 is further associated with the prechylomicron transport vesicle complex, playing a role in its movement from the endoplasmic reticulum to the Golgi apparatus, where additional lipids and apolipoproteins are incorporated [32]. The importance of CD36 in chylomicron formation has been highlighted by studies in CD36-deficient mice, which had reduced chylomicron production and an increased presence of smaller, remnant-like particles [33,34]. Beyond enterocytes, CD36 is expressed in several EEC populations, including CCK-producing I cells and other hormone-secreting EECs. The functional role of CD36 has been most extensively characterized in CCK-producing cells. In these cells, CD36 contributes to hormone release in response to luminal lipids, since CD36 knock-out led to diminished secretion of CCK and a failure to reduce food intake following lipid exposure in the duodenum [35]. Although CD36 expression has been detected in other EEC populations, direct evidence linking CD36 to the regulation of hormone secretion in these cells remains limited. Given its suggested roles in both intestinal fatty acid uptake and lipid-induced hormone secretion, particularly in CCK-producing cells, CD36 may represent an important molecular connection at the interface between nutrient absorption and satiety signaling. Furthermore, its involvement in chylomicron assembly and trafficking suggests that intracellular lipid processing may be an important component of intestinal lipid-sensing mechanisms.

### Importance of chylomicron formation

The importance of chylomicron formation in the stimulation of EECs remains a matter of debate. Several studies have reported that chylomicrons or chylomicron-associated lipid processing can enhance the secretion of GLP-1, GIP, and CCK in human and murine duodenal cultures [36–38]. By contrast, other findings in mice indicated that chylomicrons alone were insufficient to stimulate CCK release unless lauric acid (C12) was present [39]. Furthermore, the observation that several FFARs were predominantly located on the basolateral membrane of EECs indicates that fatty acids must first be absorbed before activating GPCRs to trigger hormone release. Supporting this, FFAR1 agonists stimulated GLP-1 secretion in the isolated perfused rat small intestine only when administered via the vascular side, not from the intestinal lumen [10].

Evidence from human studies indicated that different steps are required for intestinal hormone secretion in response to lipid sensing. For instance, disruption of lipid digestion with the lipase inhibitor orlistat in human volunteers reduced triglyceride hydrolysis and attenuated postprandial GLP-1 and GIP secretion, indicating that efficient lipid digestion is necessary for normal gastrointestinal hormone release [40–42]. However, another clinical study observed an enhancement of GLP-1 release in response to orlistat in obese patients with type 2 diabetes, likely due to increased delivery of undigested lipids to more distal intestinal segments [43]. In parallel, inhibition of chylomicron formation with Pluronic L-81 in rats blunted the CCK response to duodenal lipid infusion and significantly reduced GLP-1 and GIP secretion [38], highlighting the importance of intracellular lipid processing and lipoprotein assembly.

One proposed mechanism is that chylomicrons are released into the basolateral space of the intestinal epithelium, where they can be hydrolyzed by local lipases (including potentially lipoprotein lipase) into free fatty acids and monoacylglycerols. These metabolites may then activate FFARs and GPR119 located on the basolateral membrane of the EECs, thereby promoting hormone release. However, further *in vivo* studies are needed to clarify the precise contribution of chylomicron formation to this process. Furthermore, some studies suggested that CCK may directly regulate enterocyte lipid processing or chylomicron production, as mice lacking CCK exhibited impaired triglyceride absorption that cannot be fully explained by reduced postprandial pancreatic enzyme secretion alone and may also involve reduced bile delivery to the intestine [44].

Together, these observations suggest that lipid-induced hormone release depends not only on luminal fatty acids but also on their absorption and subsequent chylomicron formation, supporting a key role for post-absorptive signaling mechanisms.

### Importance of bile salts for lipid absorption

Bile acids are amphipathic, steroid-derived molecules released into the upper small intestine, where they facilitate lipid digestion and absorption. They are important regulators of lipid sensing, as they influence fatty acid uptake and the formation and size of micelles, which can in turn affect hormone secretion. For instance, food intake, and in particular fat consumption, stimulated the secretion of bile acids into the upper small intestine by CCK-mediated contraction of the gallbladder in mice [45]. However, a recent study disrupting CYP7A1 in mice, an enzyme involved in bile acid synthesis from cholesterol, reported a reduction of the bile acid pool by approximately 50%, yet paradoxically enhanced postprandial secretion of GLP-1 and PYY following refeeding [46], which was linked to a partial protection from diet-induced obesity. One possible explanation is that reduced bile acid availability impairs lipid absorption in the proximal intestine, allowing more lipids to reach the distal gut. There, either the increased local absorption of lipids or microbial metabolism may generate signals that stimulate L cells and increase circulating GLP-1 and PYY levels. However, this effect was not replicated by inhibition of lipid digestion with orlistat, where TGs remain undigested and are sequestered within the intestinal lumen, limiting their interaction with the EECs and thereby failing to stimulate hormone release. Additionally, the same study hypothesized that alterations in bile acid pools might shift intestinal lipid handling, highlighting that, depending on the bile acids present in the pool, the absorption of unsaturated over saturated fatty acids may be favored, modulating incretin secretion and satiety signaling [46].

Overall, bile acids contribute to metabolic regulation not only by enabling fat digestion but also by directly stimulating the release of gut hormones, including GLP-1, GIP, and PYY, through GPBAR1. This mechanism has been investigated in intestinal primary cultures and Ussing chamber-mounted intestinal tissues from wild-type and *Gpbar1* knock-out mice [47]. Dysregulation of bile acid pools and receptor signaling pathways contributes to metabolic disorders such as type 2 diabetes and obesity by impairing insulin sensitivity and energy balance [48–50]. In the gastrointestinal tract, dysregulated bile acid pools and their interactions with the gut microbiota can lead to changes in bile acid composition that impair intestinal barrier integrity and promote inflammation [51,52]. Additionally, excessive delivery of bile acids to the colon, as observed in bile acid malabsorption, can disrupt fluid balance and intestinal function [53,54]. Increased colonic bile acid exposure activated enteroendocrine L cells, promoting the secretion of hormones such as PYY [16]. Furthermore, recent evidence indicates that bile acids can stimulate the release of INSL5 from distal colonic L cells, as elevated circulating INSL5 levels have been observed in patients with bile acid diarrhea and were found to correlate with disease severity [55].

### Importance of acyl-CoA long chain synthetase enzymes

The initial phase of intestinal fatty acid absorption involves the acylation of fatty acids to acyl-CoA by the acyl-CoA long chain synthetase (ACSL) enzymes. ACSL5 is abundantly expressed in the small intestinal epithelium where it is the major ACSL isoform, contributing approximately 80% of total ACSL activity. ACSL5 normally facilitates fatty acid activation and triglyceride synthesis in the intestine [56]. Whilst fecal excretion of dietary lipid was not increased in intestinal-selective ASCL5 knockout mice fed a high-fat diet, postprandial GLP-1 and PYY were significantly elevated in these mice, leading to a reduction in food intake and partial protection from diet-induced obesity, consistent with an increased delivery and absorption of FFA to the more distal small intestine and suggesting potential therapeutic targeting for metabolic disease [57].

## Moleolecular mechanisms of fatty acid sensing by EECs

**Figure 2:**
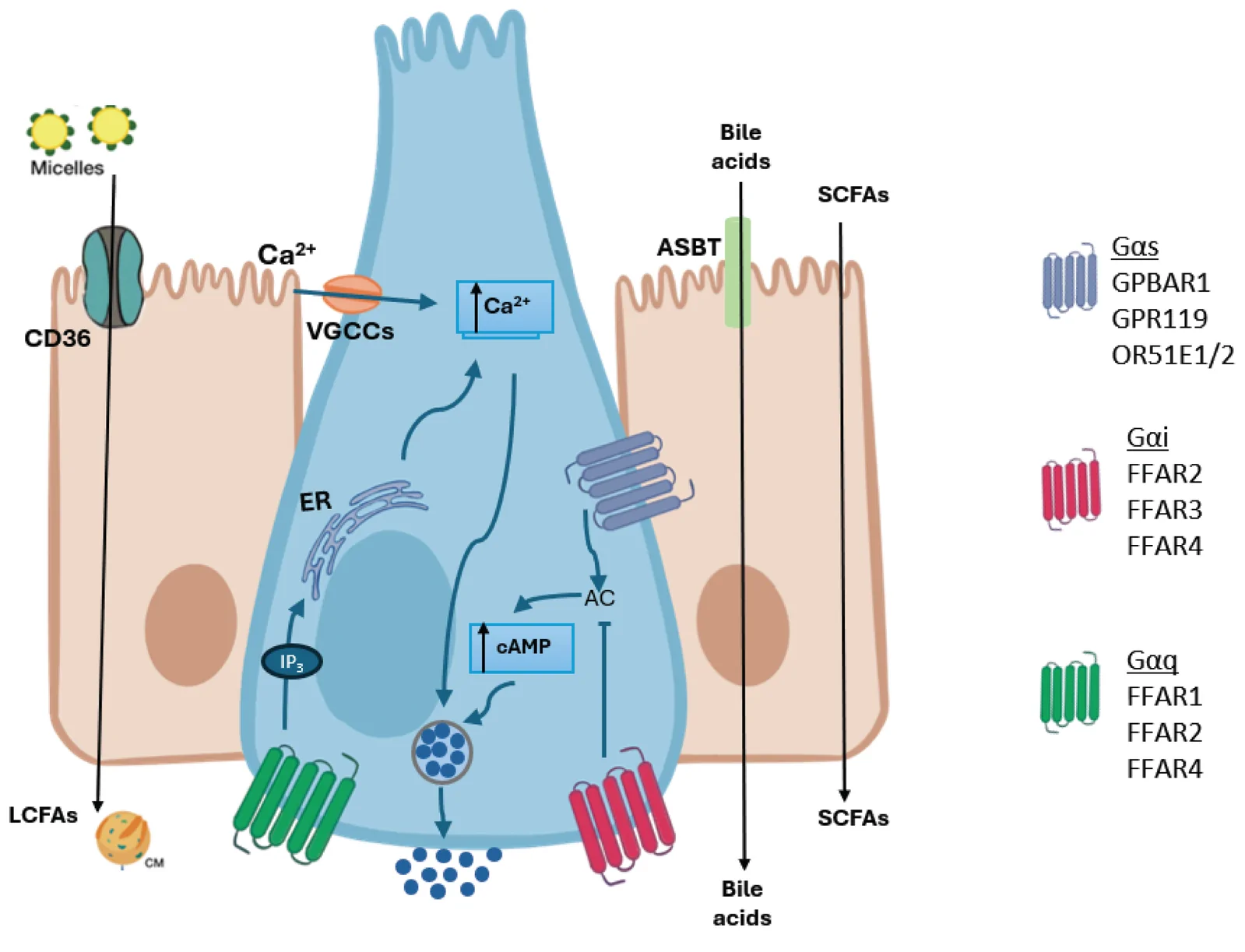
Lipid-sensing mechanisms regulating enteroendocrine hormone secretion Dietary fatty acids and bile acids activate EECs through apical and basolateral nutrient-sensing mechanisms. Fatty acids and bile acids are absorbed across the brush border by specific transporters, including CD36 and the apical sodium-dependent bile acid transporter (ASBT), before interacting with intracellular or basolateral receptors. Particularly deconjugated bile acids (products of luminal microbiota processing) can also diffuse passively across the epithelium. LCFAs activate the Gαq-coupled receptors FFAR1 and FFAR4, whereas short-chain fatty acids stimulate FFAR2 (Gαi/q), FFAR3 (Gαi), and the olfactory receptors OR51E1/OR51E2 (Gαs). Monoacylglycerols activate the Gαs-coupled receptor GPR119, while bile acids activate the Gαs-coupled receptor GPBAR1 (TGR5). Gαq signaling stimulates phospholipase C, leading to IP_3_ production, Ca^2+^ release from the endoplasmic reticulum, activation of voltage-gated calcium channels (VGCCs), and hormone exocytosis. Gαs-coupled receptors activate adenylyl cyclase, increasing intracellular cAMP and promoting hormone secretion, whereas Gαi-coupled receptors inhibit adenylyl cyclase and reduce cAMP production. Abbreviations: adenylyl cyclase (AC), ASBT, cyclic adenosine monophosphate (cAMP), cluster of differentiation 36 (CD36), endoplasmic reticulum (ER), free fatty acid receptors 1–4 (FFAR1-4), G protein-coupled bile acid receptor 1 (GPBAR1), G protein-coupled receptor (GPCR), G protein-coupled receptor 119 (GPR119), inositol 1,4,5-trisphosphate (IP3), olfactory receptors 51E1 and 51E2 (OR51E1/2), short-chain fatty acids (SCFAs), LCFAs, VGCCs.

Free fatty acids are detected by a range of GPCRs that differ in their ligand specificity (Figure 2). Among these, FFAR1 (GPR40) and FFAR4 (GPR120) primarily respond to MCFAs and LCFAs, whereas FFAR2 (GPR43) and FFAR3 (GPR41) are activated by SCFAs.

FFAR1 is responsive to both saturated and unsaturated fatty acids, with particularly high sensitivity to unsaturated species such as omega-3 and omega-6 fatty acids, including oleic acid [58]. In contrast, some studies suggest that FFAR4 appears to show greater selectivity for unsaturated LCFAs, including linoleic, α-linolenic and palmitoleic acids [25,59].

SCFA sensing in the distal intestine is mediated by FFAR2 and FFAR3, and by GPR109A (hydroxycarboxylic acid receptor HCAR2), which contribute to the detection of microbiota-derived metabolites such as acetate, propionate, and butyrate [60]. Additional receptors expand the range of fatty acid sensing, such as GPR84, activated by MCFAs [61].

Beyond classical FFARs, other receptor families are also implicated in fatty acid detection. Olfactory receptors have been identified in human and mouse gut. OR51E1 and OR51E2 (mouse homologues, Olfr558 and Olfr78) are olfactory receptors responsive to carboxylic acids of chain lengths C4–C14 and C2–C3, respectively. OR51E2/Olfr78 is primarily activated by acetate and propionate, whereas OR51E1/Olfr558 is more responsive to isovalerate and butyrate, although it can also detect longer-chain fatty acids such as nonanoic acid [62,63].

Monoacylglycerols, generated alongside free fatty acids during TG digestion, are detected by GPR119. This receptor can act in concert with FFAR1 in EECs, with evidence showing synergistic stimulation of GLP-1 secretion when both the Ca^2+^-(FFAR1) and cAMP-(GPR119) elevating pathways are activated [64].

Bile acids contribute to lipid sensing through activation of GPBAR1 (TGR5), a Gs-coupled receptor highly expressed in EECs, highlighting their dual role as both digestive agents and signaling molecules.

Although this review focuses on lipid sensing, EECs are exposed to complex nutrient mixtures rather than individual macronutrients. Single-cell transcriptomic analyses in mouse intestinal tissue and human intestinal organoids revealed that individual EECs co-express multiple nutrient-sensing receptors and transporters, providing the molecular basis for the integration of lipid, carbohydrate, and protein signals [14,15,21]. Smith et al. [23] functionally validated these findings using single-cell calcium imaging in mouse ileal organoids. Individual L cells exhibited calcium responses to glucose, the FFAR1 agonist AM1638, and tryptophan, demonstrating the pluri-responsive nature of EECs. Accordingly, nutrient-sensing pathways converge on common intracellular signaling networks, including Ca^2+^, cAMP, and ERK signaling, enabling EECs to integrate inputs from multiple macronutrients and generate coordinated hormonal responses to mixed meals. Nevertheless, because dietary lipids are the primary focus of this review, the following sections examine the molecular mechanisms of fatty acid sensing in the major EEC populations, including K, L, I, X/A and EC cells, with emphasis on the regulation of GIP, GLP-1, CCK, ghrelin and serotonin secretion, respectively.

### Fatty acid sensing by K cells and L cells

Fat ingestion increases circulating plasma GIP and GLP-1 levels in humans, rats, and mice [65–67]. This response is influenced by fatty acid chain length and composition, with LCFAs playing a dominant role. The spatial distribution of EECs underlies distinct roles in lipid sensing, with proximal K cells secreting GIP in response to dietary fat early during intestinal absorption, whereas distal L cells predominantly release GLP-1 as nutrients reach the ileum, a response that is further enhanced following bariatric surgery or delayed lipid absorption [2]. In humans, oral TG intake elicits a greater rise in plasma GIP than LCFAs alone, whereas glycerol and medium-chain TG had little effect on GIP secretion [68]. By contrast, GLP-1 secretion appears less sensitive to lipid structure, as similar increases were observed following ingestion of C8-based dietary oil and olive oil [69], which is considered to reflect the greater importance of GPR119 for GLP1 secretion (see below). Fatty acid saturation further modulates hormone release in healthy humans. Olive oil, which is rich in monounsaturated fatty acids, induced a more pronounced elevation in plasma GIP and GLP-1 than butter, which is predominantly composed of saturated fatty acids [70]. Evidence from obese mice supported this, with unsaturated LCFAs such as oleic, linoleic and linolenic acids robustly stimulating GIP secretion, whereas propionic and capric acids (C10) were ineffective [71]. Similarly, GLP-1 levels rise during fat ingestion, with stronger responses observed following monounsaturated fatty acid-rich meals compared with polyunsaturated or saturated fatty acid-based meals [72]. Another study pointed out that MCFAs could stimulate GLP-1 secretion via GPR84 in mice [61]. Pharmacological modulation can also influence lipid-induced hormone release. For example, metformin alters intestinal lipid sensing and hormone secretion: while lipid infusion increases GLP-1 and PYY levels under both chow and high-fat diet conditions, GIP secretion is reduced in high-fat-fed animals. Notably, metformin administration restored GIP release in this context, suggesting an enhancement of upper small intestinal lipid sensing pathways that may contribute to the regulation of feeding behavior [73].

At a molecular level, transcriptomic analyses reveal high expression of the free fatty acid receptors *FFAR1* and *FFAR4* in both human and murine K cells and L cells [23,74,75], supporting a role for fatty acids in cell signaling. However, their precise contribution to lipid-induced GIP and GLP-1 secretion is not fully defined. In mouse primary cell cultures, deletion of *Ffar4* partially reduced lipid-stimulated hormone secretion, an effect that was amplified when combined with *Ffar1* deficiency [64,76]. Some *in vivo* studies indicated that mice lacking either Ffar1 or Ffar4 exhibited reduced GIP secretion following oral administration of corn or lard oil [77,78], and *Ffar1/4* double knockout appeared to further impair lipid-stimulated GIP secretion [79], although double knockouts were not directly compared with *Ffar1* knockouts in the latter publication. These findings support complementary roles for FFAR1 and FFAR4 in mediating lipid-induced activation of K cells and highlight the importance of fatty acid sensing in regulating early postprandial GIP release.

Furthermore, Hirasawa et al. reported that whereas mice lacking Ffar4 exhibited reduced GLP-1 secretion following fat ingestion, *Ffar1* knockout mice showed impaired GIP and GLP-1 responses to high-fat feeding [59]. Another study showed that lipid administration stimulated the release of CCK, GIP and GLP-1 *in vivo*, but that only CCK and GIP responses were diminished in *Ffar4* knockout mice, with GLP-1 responses remaining unaffected in these animals. Importantly, exogenous CCK restored GIP secretion in *Ffar4* knockout mice, likely through normalization of gallbladder contraction and bile acid release [78], suggesting that the reduction in GIP secretion observed in *Ffar4*-deficient mice appears to be indirect.

Chylomicrons have been shown to stimulate GLP-1 and GIP secretion in both human and murine duodenal cultures [37]. Triglyceride re-esterification, and hence chylomicron formation, was impaired in mice deficient in *Mgat2* or *Dgat1*, and these mice showed reduced GIP secretion upon oral administration of TG. By contrast, increases in plasma GLP-1 and PYY in both knockout mouse strains were comparable to those observed in wild-type mice, likely reflecting a shift in lipid absorption to the more distal small intestine, where GLP-1 and PYY are expressed at higher levels. By contrast, a high dose of orlistat completely inhibited the rise in GIP, GLP-1 and PYY levels after oral triglyceride loading [80]. Together, these findings may indicate that lipid-induced GIP release depends on the digestion, absorption, and intracellular processing of dietary fats, processes closely linked to chylomicron formation. However, it remains unclear whether the critical re-esterification step occurs within EECs themselves or in neighboring enterocytes, which may facilitate lipid signaling to the basolateral surface of K cells and L cells where receptors such as FFAR1 are thought to reside.

In addition to free fatty acid signaling, the monoacylglycerol receptor GPR119, a Gαs-coupled GPCR, is an important component of lipid sensing in EECs, complementing free fatty acid signaling pathways. Activation of Gpr119 with its specific agonist AR231453 increased circulating GLP-1 and GIP levels in wild-type mice, an effect that was absent from *Gpr119* knockout animals, confirming receptor specificity [81]. Furthermore, some studies have supported a role for GPR119 in nutrient-induced hormone secretion. *Gpr119-*deficient mice showed a markedly reduced GIP response to oral lipid administration, similar to observations in *Ffar1* knockout mice, suggesting that both receptors contribute to lipid sensing in K cells [64]. By contrast, GLP-1 secretion in response to olive oil appears to rely more strongly on *Gpr119*, as it was found to be significantly reduced in *Gpr119* knockout mice but largely preserved in *Ffar1*- or *Ffar4*-deficient animals [64]. A more pronounced role for GPR119 in L cell secretion is consistent with a major reduction in the GLP-1 response to a lipid gavage in L cell-elective *Gpr119* knockout mice [82]. Therefore, the contribution of GPR119 seems to be context-dependent, where lipid-induced GIP secretion is impaired in mice lacking either *Ffar1* or *Gpr119* (but not *Ffar4*), and *Gpr119* deficiency predominantly affects GLP-1 responses, particularly under high-fat diet conditions [83].

Bile acids regulate incretin secretion through both luminal and postabsorptive mechanisms, largely involving the Gs-coupled receptor GPBAR1. Whilst intraluminal bile acids increase GLP-1 and GIP in animal models, human data suggest a more selective effect, enhancing GLP-1 but not consistently stimulating GIP in response to chenodeoxycholic acid [45,84]. Bile duct ligation in mice abolished the GIP response to an oral lipid challenge [28], likely reflecting the complex interplay between bile acids, lipid digestion, gut hormone secretion, and gastric emptying. In perfused gut and cellular systems, basolateral exposure to bile acids elicited stronger GLP-1 secretion than apical exposure. Furthermore, blocking the ASBT reduced apical bile acid–induced secretion, whereas responses to basolaterally applied bile acids remained intact, supporting a model in which bile acids must be absorbed to access basolateral GPBAR1 receptors on EECs [85]. Consistently, sevelamer, a phosphate binder that also binds bile acids, blunted GLP-1, GIP, PYY and neurotensin secretion by limiting bile acid availability and absorption [86]. Similarly, *Gpbar1* knockout models showed impaired hormone release, confirming involvement of this receptor.

Mixed bile acids robustly stimulated GLP-1 and GIP *ex vivo*, but this effect disappeared when bile acids were bound or their absorption prevented [45]. For GIP, bile acids appeared to act indirectly by facilitating lipid digestion and signaling. Their presence enhanced fatty acid–induced GIP secretion, potentially involving FABP5, which in the gut epithelium is particularly highly expressed in K cells, with global *Fabp5* knockout mice displaying impaired GIP responses to oral fat administration [28,87]. Disruption of bile flow or signaling markedly reduced lipid-induced GIP responses. Finally, bile acid distribution along the intestine is critical. ASBT inhibition shifts bile acids into the colon, where microbial metabolism deconjugates taurine and glycine from bile acids and generates secondary bile acids that can passively diffuse and activate basolateral GPBAR1 on local L cells, increasing GLP-1 (plus PYY and INSL5) release. However, chronic sequestration or depletion of bile acids may reduce overall signaling capacity. Overall, bile acid–induced incretin secretion depends on their absorption, basolateral receptor access, and interactions with lipid digestion, rather than solely on direct luminal receptor activation.

Among the olfactory receptors investigated to date, OR51E1 is one of the best-characterized in the context of gut hormone secretion and is expressed in human L cells (cell line NCI-H716). Activation of OR51E1 by nonanoic acid stimulated GLP-1 and PYY secretion in the human EEC line NCI-H716, and this response was attenuated following OR51E1 knockdown [88]. However, transcriptomic analyses of human ileal organoid-derived L cells identified OR51E2, rather than OR51E1, as the predominant olfactory receptor enriched in this cell population, although OR51E1 transcripts were also detected [89]. Similarly, OR51E2 was enriched in human K cells, whereas OR51E1 was expressed but not specifically enriched [74]. To date, however, no studies have demonstrated that activation of a specific olfactory receptor directly stimulates GIP secretion from human K cells.

Cannabinoid receptor 1 (CB1), encoded by *Cnr1*, is highly expressed in small-intestinal K cells and expressed at lower levels in L cells. Consistent with its predominantly Gi-coupled signaling, activation of CB1 by anandamide inhibited GIP, but not GLP-1, release in murine primary cultures and in rats following oral glucose administration [90]. Although these findings suggest a potential role for peripheral CB1 signaling in the regulation of incretin secretion, the physiological relevance of this pathway in humans remains unclear, particularly given the well-established role of central CB1 receptors in the regulation of appetite and energy homeostasis.

## L cells in the large intestine

Recent studies indicate that SCFAs stimulate gut hormone secretion through activation of ORs and FFARs. FFAR2 and FFAR3 are expressed in colonic enteroendocrine L cells, where they play key roles in energy homeostasis by regulating the secretion of PYY and GLP-1 [91]. Secretion of those hormones was reduced in primary endocrine cells derived from *Ffar2* and *Ffar3* knockout mice [60,92], and colonization of germ-free mice increased PYY levels in wild-type but not *Ffar3*-deficient animals, demonstrating that gut microbiota-derived SCFAs regulate PYY secretion via Ffar3. In addition to FFARs, ORs also contribute to this process. Acetate promoted PYY secretion in STC-1 cells through the Olfr78–protein kinase A signaling pathway, and this effect was attenuated by *Olfr78* knockdown [93]. Similarly, fructo-oligosaccharide intake increased colonic SCFA levels and plasma PYY concentrations in wild-type mice but not in *Olfr78* knockout mice, supporting a role for Olfr78 in mediating SCFA-induced PYY secretion *in vivo* [93]. These findings suggest that Olfr78 functions as a microbial metabolite sensor linking gut microbiota-derived SCFAs to enteroendocrine hormone release. This concept was further supported by studies demonstrating that other ORs responded to fatty acids of different chain lengths. For instance, OR51E2, highly expressed in the human gut, was activated by palmitic acid [94], while OR51E1 sensed a range of MCFAs, including nonanoic, C10, and octanoic acids, and stimulated PYY and GLP-1 secretion along with ERK phosphorylation and cAMP production in human enteroendocrine L cell line NCI-H716 [88]. Furthermore, the dicarboxylic azelaic acid increased plasma GLP-1 levels in mice fed a high-fat diet via Olfr554 activation, an effect abolished in receptor-deficient models [95]. Collectively, these findings demonstrate that SCFAs and some MCFAs regulate gut hormone secretion via OR- and FFAR-mediated pathways, highlighting the importance of those receptors in appetite control and metabolic regulation.

### Fatty acid sensing by I cells

Fatty acids stimulate CCK secretion in both mice and humans, primarily through GPR119 and FFAR1 receptors. In STC-1 cells, LCFA induced CCK release by acting on Ffar4 [96]. LCFAs also increased intracellular Ca^2+^ levels and triggered CCK secretion partially through Ffar1, since this response was reduced, but not completely eliminated, in *Ffar1* knockout mice [97]. In humans, intragastric and intraduodenal administration of C12 fatty acids produced a greater rise in plasma CCK levels than C10, consistent with the preference of FFAR1 for LCFAs [58,66,67]. Similarly, oral intake of olive oil increased circulating CCK, whereas C4-dietary oil (a prodrug for 2-mono-oleoylglycerol) did not, highlighting the importance of LCFA signaling beyond monoacylglycerols alone [98]. In mouse models, deletion of *Ffar1* significantly reduced CCK responses to olive oil, and both *Ffar1*- and *Ffar4*-deficient mice showed diminished CCK levels following corn oil administration [78,97]. Moreover, linolenic acid–induced Ca^2+^ signaling and CCK secretion in purified I cells were abolished in the absence of Ffar1, underscoring its central role in LCFA sensing [97].

CD36 has been reported to be involved in CCK secretion, as *Cd36* knockout mice exhibited reduced CCK secretion, with calmodulin kinase II participating in downstream signaling, supporting the role of chylomicron formation and basolateral sensing in this pathway [35]. Because lipid absorption is essential for triglyceride-induced CCK secretion, additional sensing mechanisms have been proposed. One candidate is the immunoglobulin-like domain-containing receptor 1 (ILDR1), identified as a potential lipoprotein sensor. In Ildr1-deficient mice, plasma CCK responses to oral fatty acids were abolished [39]. In isolated intestinal CCK cells, high-density lipoprotein (HDL) combined with fatty acids increased intracellular Ca^2+^ in wild-type but not *Ildr1*-deficient mice. The authors suggested a mechanism in which HDL produced by enterocytes interacts with ILDR1 on the basolateral membrane to stimulate CCK release, although the precise role of ILDR1 remains unclear [39].

CB1 xpression has been detected in CCK-producing cells, and the endocannabinoid arachidonoyl glycerol has been reported to stimulate CCK release from STC-1 cells through CB1 activation [99]. However, the physiological relevance of CB1 signaling in human EECs remains unclear. CB1 is primarily a Gi-coupled receptor, and the well-established effects of the endocannabinoid system on appetite and energy balance are largely attributed to CB1 signaling within the central nervous system (CNS). Whilst, clinical and pharmacological studies showed that CB1 agonists increase appetite and food intake, whereas CB1 antagonists such as Rimonabant have been shown to reduce appetite and body weight in humans, these effects are largely attributed to CB1 receptors expressed in the CNS [100,101].

In addition to endocrine signaling, intestinal lipid sensing engages neural pathways, particularly via the vagus nerve, forming a gut–brain axis that is essential for appetite regulation. Lipid-induced activation of EECs stimulates vagal afferent fibers either directly or indirectly through hormone release [102,103]. Particularly, CCK relays signals of nutrient intake to the brain through vagal afferents, suppressing appetite and enhancing the preference for nutritious sweet and fatty foods [104].

### Fatty acid sensing by X/A cells

Ghrelin exists in two forms: acylated (fatty acid–modified) ghrelin and des-acyl (unacylated) ghrelin. The acyl modification is essential for binding to its receptor, the growth hormone secretagogue receptor [105]. MCFAs derived from dietary medium-chain triglycerides have been considered a primary source for this modification [106,107]. However, although researchers suggest that the source of MCFAs used for ghrelin modification originates from the diet, a contribution of endogenous sources has also been suggested. Bando et al. demonstrated that LCFAs can serve as precursors for ghrelin acylation in a ghrelinoma cell line (MGN3-1) [108]. In ghrelin-producing cells, LCFAs are converted into medium-chain acyl-CoA through β-oxidation before being transported from mitochondria or peroxisomes to the cytoplasm via enzymes such as acyl-CoA thioesterases (ACOTs) and carnitine octanoyltransferase [109]. Indeed, recent studies further indicated that ghrelin-producing cells can generate the required MCFAs internally through β-oxidation of circulating LCFAs, as neither fat-free diets nor removal of intestinal microbiota reduced acyl-ghrelin levels in mice [110]. *In vitro* findings from pancreas-derived ghrelinoma (PG-1) cells showed expression of several ACOT genes (Acot2, 4, 6, 8, 9, 10 and 13), supporting the idea that ghrelin-producing cells can synthesize MCFAs from LCFAs and very-long-chain fatty acids [109]. Thus, while dietary MCFAs can contribute to ghrelin acylation, these cells are also capable of utilizing endogenous lipid sources independently of food intake [109]. The enzyme ghrelin O-acyltransferase (GOAT) is responsible for attaching MCFAs to ghrelin [111]. GOAT (*Mboat4*) knockout mice showed reduced weight gain, highlighting its potential as a therapeutic target for conditions related to energy imbalance and gastrointestinal dysfunction [111].

Fatty acid sensing in ghrelin cells involves several proteins, including CD36, α-gustducin, and GPCRs such as FFAR1 and FFAR4 [110]. Additionally, α-gustducin was implicated in the regulation of ghrelin octanoylation *in vivo* in mice, and it was suggested that ghrelin-producing cells can directly sense medium- and long-chain fatty acids via receptors such as FFAR4 [112,113]. However, the effects of dietary fat on ghrelin secretion remain controversial: while some studies indicated that fat intake may elevate ghrelin levels, others demonstrated that fat consumption suppressed circulating ghrelin concentrations or produced only modest inhibitory effects compared with other macronutrients [114–116].

### Fatty acid sensing by EC cells

ECs in the gastrointestinal tract are responsible for approximately 90%–95% of the body’s serotonin (5-HT) production. EC cells in human duodenal organoids express several GPCRs involved in nutrient sensing, including FFAR2, FFAR4, GPR119, and olfactory receptors such as OR51E1 and OR51E2 [75]. Among fatty acid receptors, FFAR1 was, however, present at very low levels in murine EC cells, whereas FFAR4 showed higher expression but was not significantly enriched compared with the surrounding epithelium, likely dominated by enterocytes. By contrast, FFAR2 and OR51E1 were highly expressed and enriched [117]. Olfactory receptors OR51E1/Olfr558 and OR51E2/Olfr78 have been identified in EC cells, in human duodenal organoids [75], and in mouse distal intestine, where microbial density is highest [63]. *Olfr78* is strongly expressed in the mouse colon, although this pattern is less pronounced in humans. Microbial metabolites, particularly SCFAs such as acetate, propionate and butyrate, directly stimulated murine EC cells, increasing serotonin production by promoting its release and up-regulating tryptophan hydroxylase 1 (TPH1) expression [118,119]. At the signaling level, FFAR2 primarily mediated Ca^2+^ responses to acetate and butyrate, whereas OR51E1 was associated with cAMP responses to butyrate and isovalerate. Because OR51E1 did not respond to acetate, OR51E2 has been proposed as a candidate mediator of the acetate-induced cAMP response, although direct evidence is still lacking. Among these metabolites, studies using murine intestinal organoids showed that isovalerate elicited the strongest activation of “EC cells”, while butyrate and isobutyrate had more modest effects [63]. Beyond SCFAs, other microbiota-derived compounds also modulated serotonin pathways. For example, butyrate and propionate enhanced 5-HT release and TPH1 expression *in vitro*, while the secondary bile acid deoxycholate increased serotonin levels in the colon and influenced motility via the GPBAR1 receptor expressed on EC cells [118–120].

## Clinical perspective

From a clinical perspective, intestinal lipid sensing is a crucial mechanism by which the gut detects dietary fats and coordinates digestive, hormonal, and metabolic responses. When lipids are absorbed in the small intestine, they activate receptors such as FFAR1 and GPR119 on EECs, leading to the release of hormones including CCK, GLP-1, GIP and PYY. These hormones slow gastric emptying, promote satiety and/or satiation, and enhance insulin secretion.

Clinically, dysregulation of lipid digestion, absorption, intracellular trafficking and enteroendocrine lipid-sensing pathways has been associated with metabolic disorders, although most studies provide associative evidence without demonstrating causality [121]. Reduced incretin effect, particularly involving GIP and GLP-1, contributes to impaired insulin secretion and postprandial glucose intolerance [122]. Insulin-resistant states are associated with exaggerated postprandial lipemia and increased intestinal lipoprotein production, including ApoB48-containing particles, reflecting altered handling of dietary lipids [123]. GLP-1 receptor agonists such as semaglutide and liraglutide improve glycemic control, promote weight loss, reduce cardiovascular risk [124,125], and also reduce postprandial triglycerides and ApoB48-containing lipoproteins, consistent with modulation of intestinal–pancreatic axis signaling [126–130]. However, the mechanisms by which GLP-1 regulates lipid metabolism are incompletely understood. Recent studies have shown that gut-targeted agonism of FFAR1 and GPR119 (named K-757 and K-833, respectively) induced robust secretion of GLP-1, PYY, CCK and GIP in both preclinical models and humans, highlighting the therapeutic potential of intestinal nutrient-sensing pathways [131–133]. However, the effects on body weight were modest, suggesting that activation of endogenous enteroendocrine signaling alone may be insufficient to replicate the magnitude of weight loss achieved with GLP-1 receptor agonists [132,133].Additional clinical evidence showed drugs that directly alter lipid digestion and gut hormone secretion. Orlistat reduces the hydrolysis of dietary triglycerides and consequently modifies the generation of luminal lipid-derived signals that stimulate EECs [134]. Studies by Beglinger et al. reported that inhibition of fat digestion attenuated postprandial secretion of GLP-1 and PYY, highlighting the dependence of enteroendocrine responses on the formation of absorbable lipid metabolites [40]. Bile acids play a critical role in coupling lipid absorption to hormone secretion through activation of GPBAR1. Different studies showed that bile acids stimulated GLP-1 secretion via GPBAR activation in EECs. Bile acid sequestrants can alter bile acid signaling and potentially enhance endogenous GLP-1 secretion and glycemic control [135–137]. Although the metabolic effects of these agents are less pronounced than those achieved with GLP-1 receptor agonists, they provide important clinical suggestion that manipulation of intestinal lipid processing and nutrient-sensing pathways can influence EEC function and whole-body metabolic homeostasis.

Bariatric surgery alters intestinal lipid sensing by modifying the site and kinetics of nutrient delivery along the gastrointestinal tract. Following procedures such as Roux-en-Y gastric bypass, dietary lipids are delivered more rapidly to the more distal small intestine and are only digested here, as bile and pancreatic enzymes are delivered more distally, increasing the exposure of distal EECs to nutrients and bile acids. This altered nutrient flow has been associated with enhanced postprandial secretion of gut hormones, particularly GLP-1 and PYY, although the magnitude of these responses varies among individuals and surgical procedures, and their relative contribution to the metabolic benefits of bariatric surgery remains under investigation [138,139]. Current evidence suggests that the metabolic benefits of bariatric surgery are likely to arise from a combination of altered nutrient delivery, bile acid signaling, intestinal adaptation, and changes in enteroendocrine function, rather than from lipid malabsorption [140,141].

Despite significant advances, several challenges and gaps remain in our understanding of intestinal lipid sensing. The relative contributions of different lipid species, receptor subtypes, and intestinal regions to overall metabolic regulation are not fully elucidated. Moreover, emerging evidence also suggests that the gut microbiota may modulate lipid sensing, either by altering luminal lipid composition or by influencing EEC function [142]. These interactions represent an important area for future research.

In conclusion, intestinal lipid sensing represents a complex physiological process that plays a central role in regulating metabolism and energy balance. Following hydrolysis by pancreatic lipases, lipid-derived products such as LCFAs and monoacylglycerols are absorbed by enterocytes and detected through a combination of membrane receptors, intracellular metabolic pathways and lipid transport systems. Lipid sensors such as FFARs, GPR119, GPBAR and ORs are expressed in EECs and contribute to nutrient-stimulated hormone secretion, including GLP-1, GIP, CCK and PYY. These hormonal signals coordinate gastric emptying, satiety/satiation, insulin secretion and postprandial lipid handling. Collectively, intestinal lipid sensing provides a mechanistic link between dietary fat exposure and systemic metabolic responses, and its dysregulation is increasingly recognized as contributing to metabolic disease.

## Open Access

Open access for this article was enabled by the participation of the University of Cambridge in an all-inclusive Read & Publish agreement with Portland Press and the Biochemical Society under an agreement with JISC.

## Abbreviations

ACSL: acyl-CoA long chain synthetase
ACOTs: acyl-CoA thioesterases
ASBT: apical sodium-dependent bile acid transporter
apoB: apolipoprotein B
CB1: Cannabinoid receptor 1
CNS: central nervous system
CCK: cholecystokinin
cAMP: cyclic adenosine monophosphate
ER: endoplasmic reticulum
EC: enterochromaffin cells
EECs: enteroendocrine cells
FFARs: fatty acid–sensing receptors
GLP-1: glucagon-like peptide-1
GIP: glucose-dependent insulinotropic polypeptide
GPCRs: G protein–coupled receptors
GOAT: ghrelin O-acyltransferase
HDL: high-density lipoprotein
ILDR1: immunoglobulin-like domain-containing receptor 1
OR51E1/2: olfactory receptors 51E1 and 51E2
PYY: peptide YY
pre-CMs: pre-chylomicrons
SCFAs: short-chain fatty acids
TGs: triacylglycerols
TPH1: tryptophan hydroxylase 1
VGCCs: voltage-gated calcium channels

## References

[1] Kaur N., Chugh V. and Gupta A.K. (2014) Essential fatty acids as functional components of foods—a review. J. Food Sci. Technol. 51, 2289–2303 10.1007/s13197-012-0677-0 25328170 PMC4190204

[2] Gribble F.M. and Reimann F. (2019) Function and mechanisms of enteroendocrine cells and gut hormones in metabolism. Nat. Rev. Endocrinol. 15, 226–237 10.1038/s41574-019-0168-8 30760847

[3] Harper A.A. and Raper H.S. (1943) Pancreozymin, a stimulant of the secretion of pancreatic enzymes in extracts of the small intestine. J. Physiol. 102, 115–125 10.1113/jphysiol.1943.sp004021 16991584 PMC1393423

[4] Ivy A.C. (1929) A hormone mechanism for gall-bladder contraction & evacuation. Am. J. Surg. 7, 455–459 10.1016/S0002-9610(29)90551-1

[5] Nauck M.A., Bartels E., Orskov C., Ebert R. and Creutzfeldt W. (1993) Additive insulinotropic effects of exogenous synthetic human gastric inhibitory polypeptide and glucagon-like peptide-1-(7-36) amide infused at near-physiological insulinotropic hormone and glucose concentrations. J. Clin. Endocrinol. Metab. 76, 912–917 10.1210/jcem.76.4.8473405 8473405

[6] Müller T.D., Finan B., Bloom S.R., D'Alessio D., Drucker D.J., Flatt P.R. et al. (2019) Glucagon-like peptide 1 (GLP-1). Mol. Metab. 30, 72–130 10.1016/j.molmet.2019.09.010 31767182 PMC6812410

[7] Müller T.D., Adriaenssens A., Ahrén B., Blüher M., Birkenfeld A.L., Campbell J.E. et al. (2025) Glucose-dependent insulinotropic polypeptide (GIP). Mol. Metab. 95, 102118 10.1016/j.molmet.2025.102118 40024571 PMC11931254

[8] Mawe G.M. and Hoffman J.M. (2013) Serotonin signalling in the gut–functions, dysfunctions and therapeutic targets. Nat. Rev. Gastroenterol. Hepatol. 10, 473–486 10.1038/nrgastro.2013.105 23797870 PMC4048923

[9] Higgins S.C., Gueorguiev M. and Korbonits M. (2007) Ghrelin, the peripheral hunger hormone. Ann. Med. 39, 116–136 10.1080/07853890601149179 17453675

[10] Christensen L.W., Kuhre R.E., Janus C., Svendsen B. and Holst J.J. (2015) Vascular, but not luminal, activation of FFAR1 (GPR40) stimulates GLP-1 secretion from isolated perfused rat small intestine. Physiol. Rep. 3, e12551 10.14814/phy2.12551 26381015 PMC4600392

[11] Santos-Hernández M., Reimann F. and Gribble F.M. (2024) Cellular mechanisms of incretin hormone secretion. J. Mol. Endocrinol. 72, e230112 10.1530/JME-23-0112 38240302 PMC10959011

[12] Beumer J., Artegiani B., Post Y., Reimann F., Gribble F., Nguyen T.N. et al. (2018) Enteroendocrine cells switch hormone expression along the crypt-to-villus BMP signalling gradient. Nat. Cell Biol. 20, 909–916 10.1038/s41556-018-0143-y 30038251 PMC6276989

[13] Beumer J., Puschhof J., Bauzá-Martinez J., Martínez-Silgado A., Elmentaite R., James K.R. et al. (2020) High-resolution mRNA and secretome atlas of human enteroendocrine cells. Cell 181, 1291–306.e19 10.1016/j.cell.2020.04.036 32407674

[14] Haber A.L., Biton M., Rogel N., Herbst R.H., Shekhar K., Smillie C. et al. (2017) A single-cell survey of the small intestinal epithelium. Nature 551, 333–339 10.1038/nature24489 29144463 PMC6022292

[15] Egerod K.L., Engelstoft M.S., Grunddal K.V., Nohr M.K., Secher A., Sakata I. et al. (2012) A major lineage of enteroendocrine cells coexpress CCK, secretin, GIP, GLP-1, PYY, and neurotensin but not somatostatin. Endocrinology 153, 5782–5795 10.1210/en.2012-1595 23064014 PMC7958714

[16] Billing L.J., Smith C.A., Larraufie P., Goldspink D.A., Galvin S., Kay R.G. et al. (2018) Co-storage and release of insulin-like peptide-5, glucagon-like peptide-1 and peptideYY from murine and human colonic enteroendocrine cells. Mol. Metab. 16, 65–75 10.1016/j.molmet.2018.07.011 30104166 PMC6158034

[17] Billing L.J., Larraufie P., Lewis J., Leiter A., Li J., Lam B. et al. (2019) Single cell transcriptomic profiling of large intestinal enteroendocrine cells in mice - Identification of selective stimuli for insulin-like peptide-5 and glucagon-like peptide-1 co-expressing cells. Mol. Metab. 29, 158–169 10.1016/j.molmet.2019.09.001 31668387 PMC6812004

[18] Bai L., Sivakumar N., Yu S., Mesgarzadeh S., Ding T., Ly T. et al. (2022) Enteroendocrine cell types that drive food reward and aversion. Elife 11, e74964 10.7554/eLife.74964 35913117 PMC9363118

[19] Habib A.M., Richards P., Cairns L.S., Rogers G.J., Bannon C.A.M., Parker H.E. et al. (2012) Overlap of endocrine hormone expression in the mouse intestine revealed by transcriptional profiling and flow cytometry. Endocrinology 153, 3054–3065 10.1210/en.2011-2170 22685263 PMC3440453

[20] Hayashi M., Kaye J.A., Douglas E.R., Joshi N.R., Gribble F.M., Reimann F. et al. (2023) Enteroendocrine cell lineages that differentially control feeding and gut motility. Elife 12, e78512 10.7554/eLife.78512 36810133 PMC10032656

[21] Smith C.A., Lu V.B., Bany Bakar R., Miedzybrodzka E., Davison A., Goldspink D. et al. (2025) Single-cell transcriptomics of human organoid-derived enteroendocrine cell populations from the small intestine. J. Physiol. 603, 7751–7763 10.1113/JP287463 39639676 PMC7617304

[22] Davison A., Reimann F. and Gribble F.M. (2025) Molecular mechanisms of stimulus detection and secretion in enteroendocrine cells. Curr. Opin. Neurobiol. 92, 103045 10.1016/j.conb.2025.103045 40378579

[23] Smith C.A., O'Flaherty E.A.A., Guccio N., Punnoose A., Darwish T., Lewis J.E. et al. (2024) Single-cell transcriptomic atlas of enteroendocrine cells along the murine gastrointestinal tract. PloS ONE 19, e0308942 10.1371/journal.pone.0308942 39378212 PMC11460673

[24] Beumer J., Geurts M.H., Geurts V., Andersson-Rolf A., Akkerman N., Völlmy F. et al. (2024) Description and functional validation of human enteroendocrine cell sensors. Science 386, 341–348 10.1126/science.adl1460 39418382 PMC7616728

[25] Kimura I., Ichimura A., Ohue-Kitano R. and Igarashi M. (2020) Free fatty acid receptors in health and disease. Physiol. Rev. 100, 171–210 10.1152/physrev.00041.2018 31487233

[26] Papamandjaris A.A., MacDougall D.E. and Jones P.J. (1998) Medium chain fatty acid metabolism and energy expenditure: obesity treatment implications. Life Sci. 62, 1203–1215 10.1016/S0024-3205(97)01143-0 9570335

[27] Iqbal J. and Hussain M.M. (2009) Intestinal lipid absorption. Am. J. Physiol. Endocrinol. Metab. 296, E1183–E1194 10.1152/ajpendo.90899.2008 19158321 PMC2692399

[28] Shibue K., Yamane S., Harada N., Hamasaki A., Suzuki K., Joo E. et al. (2015) Fatty acid-binding protein 5 regulates diet-induced obesity via GIP secretion from enteroendocrine K cells in response to fat ingestion. Am. J. Physiol. Endocrinol. Metab. 308, E583–E591 10.1152/ajpendo.00543.2014 25628425

[29] Xiao C., Stahel P. and Lewis G.F. (2019) Regulation of chylomicron secretion: focus on post-assembly mechanisms. Cell Mol. Gastroenterol. Hepatol. 7, 487–501 10.1016/j.jcmgh.2018.10.015 30819663 PMC6396431

[30] Raka F., Farr S., Kelly J., Stoianov A. and Adeli K. (2019) Metabolic control via nutrient-sensing mechanisms: role of taste receptors and the gut-brain neuroendocrine axis. Am. J. Physiol. Endocrinol. Metab 317, E559–E572 10.1152/ajpendo.00036.2019 31310579

[31] Nauli A.M., Nassir F., Zheng S., Yang Q., Lo C.M., Vonlehmden S.B. et al. (2006) CD36 is important for chylomicron formation and secretion and may mediate cholesterol uptake in the proximal intestine. Gastroenterology 131, 1197–1207 10.1053/j.gastro.2006.08.012 17030189 PMC1994908

[32] Siddiqi S., Saleem U., Abumrad N.A., Davidson N.O., Storch J., Siddiqi S.A. et al. (2010) A novel multiprotein complex is required to generate the prechylomicron transport vesicle from intestinal ER. J. Lipid Res. 51, 1918–1928 10.1194/jlr.M005611 20237389 PMC2882727

[33] Drover V.A., Ajmal M., Nassir F., Davidson N.O., Nauli A.M., Sahoo D. et al. (2005) CD36 deficiency impairs intestinal lipid secretion and clearance of chylomicrons from the blood. J. Clin. Invest. 115, 1290–1297 10.1172/JCI21514 15841205 PMC1074677

[34] Masuda D., Hirano K., Oku H., Sandoval J.C., Kawase R., Yuasa-Kawase M. et al. (2009) Chylomicron remnants are increased in the postprandial state in CD36 deficiency. J. Lipid Res. 50, 999–1011 10.1194/jlr.P700032-JLR200 18753675 PMC2666186

[35] Sundaresan S., Shahid R., Riehl T.E., Chandra R., Nassir F., Stenson W.F. et al. (2013) CD36-dependent signaling mediates fatty acid-induced gut release of secretin and cholecystokinin. FASEB J. 27, 1191–1202 10.1096/fj.12-217703 23233532 PMC3574291

[36] Lu W.J., Yang Q., Yang L., Lee D., D'Alessio D. and Tso P. (2012) Chylomicron formation and secretion is required for lipid-stimulated release of incretins GLP-1 and GIP. Lipids 47, 571–580 10.1007/s11745-011-3650-1 22297815 PMC3506258

[37] Psichas A., Larraufie P.F., Goldspink D.A., Gribble F.M. and Reimann F. (2017) Chylomicrons stimulate incretin secretion in mouse and human cells. Diabetologia 60, 2475–2485 10.1007/s00125-017-4420-2 28866808 PMC5850988

[38] Glatzle J., Wang Y., Adelson D.W., Kalogeris T.J., Zittel T.T., Tso P. et al. (2003) Chylomicron components activate duodenal vagal afferents via a cholecystokinin A receptor-mediated pathway to inhibit gastric motor function in the rat. J. Physiol. 550, 657–664 10.1113/jphysiol.2003.041673 12766241 PMC2343045

[39] Chandra R., Wang Y., Shahid R.A., Vigna S.R., Freedman N.J. and Liddle R.A. (2013) Immunoglobulin-like domain containing receptor 1 mediates fat-stimulated cholecystokinin secretion. J. Clin. Invest. 123, 3343–3352 10.1172/JCI68587 23863714 PMC3726170

[40] Beglinger S., Drewe J., Schirra J., Göke B., D'Amato M. and Beglinger C. (2010) Role of fat hydrolysis in regulating glucagon-like peptide-1 secretion. J. Clin. Endocrinol. Metab. 95, 879–886 10.1210/jc.2009-1062 19837920

[41] Feinle C., Rades T., Otto B. and Fried M. (2001) Fat digestion modulates gastrointestinal sensations induced by gastric distention and duodenal lipid in humans. Gastroenterology 120, 1100–1107 10.1053/gast.2001.23232 11266374

[42] Ellrichmann M., Kapelle M., Ritter P.R., Holst J.J., Herzig K.H., Schmidt W.E. et al. (2008) Orlistat inhibition of intestinal lipase acutely increases appetite and attenuates postprandial glucagon-like peptide-1-(7-36)-amide-1, cholecystokinin, and peptide YY concentrations. J. Clin. Endocrinol. Metab. 93, 3995–3998 10.1210/jc.2008-0924 18647814

[43] Damci T., Yalin S., Balci H., Osar Z., Korugan U., Ozyazar M. et al. (2004) Orlistat augments postprandial increases in glucagon-like peptide 1 in obese type 2 diabetic patients. Diabetes Care. 27, 1077–1080 10.2337/diacare.27.5.1077 15111524

[44] Lo C.M., King A., Samuelson L.C., Kindel T.L., Rider T., Jandacek R.J. et al. (2010) Cholecystokinin knockout mice are resistant to high-fat diet-induced obesity. Gastroenterology 138, 1997–2005 10.1053/j.gastro.2010.01.044 20117110 PMC3049264

[45] Kuhre R.E., Wewer Albrechtsen N.J., Larsen O., Jepsen S.L., Balk-Møller E., Andersen D.B. et al. (2018) Bile acids are important direct and indirect regulators of the secretion of appetite- and metabolism-regulating hormones from the gut and pancreas. Mol. Metab. 11, 84–95 10.1016/j.molmet.2018.03.007 29656109 PMC6001409

[46] Chan A.P., Jarrett K.E., Lai R.W., Brearley-Sholto M.C., Cheng A.S., Taveras M.O. et al. (2026) Bile acids regulate lipid metabolism through selective actions on fatty acid absorption. Cell Metab. 38, 263–280.e10 10.1016/j.cmet.2025.11.010 41386220 PMC12767720

[47] Brighton C.A., Rievaj J., Kuhre R.E., Glass L.L., Schoonjans K., Holst J.J. et al. (2015) Bile acids trigger GLP-1 release predominantly by accessing basolaterally located G protein-coupled bile acid receptors. Endocrinology 156, 3961–3970 10.1210/en.2015-1321 26280129 PMC4606749

[48] Chiang J.Y.L. (2017) Bile acid metabolism and signaling in liver disease and therapy. Liver Res. 1, 3–9 10.1016/j.livres.2017.05.001 29104811 PMC5663306

[49] Kim H. and Fang S. (2018) Crosstalk between FXR and TGR5 controls glucagon-like peptide 1 secretion to maintain glycemic homeostasis. Lab Anim Res. 34, 140–146 10.5625/lar.2018.34.4.140 30671099 PMC6333617

[50] Shapiro H., Kolodziejczyk A.A., Halstuch D. and Elinav E. (2018) Bile acids in glucose metabolism in health and disease. J. Exp. Med. 215, 383–396 10.1084/jem.20171965 29339445 PMC5789421

[51] Jia W., Xie G. and Jia W. (2018) Bile acid–microbiota crosstalk in gastrointestinal inflammation and carcinogenesis. Nat. Rev. Gastroenterol. Hepatol. 15, 111–128 10.1038/nrgastro.2017.119 29018272 PMC5899973

[52] Song G., Xie Y., Yi L., Cheng W., Jia H., Shi W. et al. (2025) Bile acids affect intestinal barrier function through FXR and TGR5. Front Med. (Lausanne) 12, 1607899 10.3389/fmed.2025.1607899 40692955 PMC12277261

[53] Barkun A.N., Love J., Gould M., Pluta H. and Steinhart H. (2013) Bile acid malabsorption in chronic diarrhea: pathophysiology and treatment. Can. J. Gastroenterol. 27, 653–659 10.1155/2013/485631 24199211 PMC3816948

[54] Camilleri M. and Nurko S. (2022) Bile acid diarrhea in adults and adolescents. Neurogastroenterol. Motil. 34, e14287 10.1111/nmo.14287 34751982 PMC8957499

[55] Bannon C.A., Walters J.R.F., Wu T., Kay R.G., Punnoose A., Spiller R.C. et al. (2026) Insulin-like peptide 5 is released in response to bile acid in the rectum and is associated with diarrhoea severity in patients with bile acid diarrhoea. Gut 75, 278–288 10.1136/gutjnl-2025-335393 40701790 PMC7618615

[56] Luo Q., Das A., Oldoni F., Wu P., Wang J. and Luo F. (2023) Role of ACSL5 in fatty acid metabolism. Heliyon 9, e13316 10.1016/j.heliyon.2023.e13316 36816310 PMC9932481

[57] Griffin J.D., Zhu Y., Reeves A., Buhman K.K. and Greenberg A.S. (2024) Intestinal Acyl-CoA synthetase 5 (ACSL5) deficiency potentiates postprandial GLP-1 & PYY secretion, reduces food intake, and protects against diet-induced obesity. Mol. Metab. 83, 101918 10.1016/j.molmet.2024.101918 38499083 PMC10990902

[58] Briscoe C.P., Tadayyon M., Andrews J.L., Benson W.G., Chambers J.K., Eilert M.M. et al. (2003) The orphan G protein-coupled receptor GPR40 is activated by medium and long chain fatty acids. J. Biol. Chem. 278, 11303–11311 10.1074/jbc.M211495200 12496284

[59] Hirasawa A., Tsumaya K., Awaji T., Katsuma S., Adachi T., Yamada M. et al. (2005) Free fatty acids regulate gut incretin glucagon-like peptide-1 secretion through GPR120. Nat. Med. 11, 90–94 10.1038/nm1168 15619630

[60] Tolhurst G., Heffron H., Lam Y.S., Parker H.E., Habib A.M., Diakogiannaki E. et al. (2012) Short-chain fatty acids stimulate glucagon-like peptide-1 secretion via the G-protein-coupled receptor FFAR2. Diabetes 61, 364–371 10.2337/db11-1019 22190648 PMC3266401

[61] Nonaka H., Ohue-Kitano R., Masujima Y., Igarashi M. and Kimura I. (2022) Dietary medium-chain triglyceride decanoate affects glucose homeostasis through GPR84-mediated GLP-1 secretion in mice. Front. Nutr 9, 848450 10.3389/fnut.2022.848450 35399667 PMC8987919

[62] Audouze K., Tromelin A., Le Bon A.M., Belloir C., Petersen R.K., Kristiansen K. et al. (2014) Identification of odorant-receptor interactions by global mapping of the human odorome. PloS ONE 9, e93037 10.1371/journal.pone.0093037 24695519 PMC3973694

[63] Bellono N.W., Bayrer J.R., Leitch D.B., Castro J., Zhang C., O'Donnell T.A. et al. (2017) Enterochromaffin cells are gut chemosensors that couple to sensory neural pathways. Cell 170, 185–198.e16 10.1016/j.cell.2017.05.034 28648659 PMC5839326

[64] Ekberg J.H., Hauge M., Kristensen L.V., Madsen A.N., Engelstoft M.S., Husted A.S. et al. (2016) GPR119, a major enteroendocrine sensor of dietary triglyceride metabolites coacting in synergy with FFA1 (GPR40). Endocrinol 157, 4561–4569 10.1210/en.2016-1334 PMC7212052 27779915

[65] Higashimoto Y., Opara E.C. and Liddle R.A. (1995) Dietary regulation of glucose-dependent insulinotropic peptide (GIP) gene expression in rat small intestine. Comp. Biochem. Physiol. C Pharmacol. Toxicol. Endocrinol. 110, 207–214 10.1016/0742-8413(94)00087-Q 7599968

[66] McLaughlin J., Grazia Lucà M., Jones M.N., D'Amato M., Dockray G.J. and Thompson D.G. (1999) Fatty acid chain length determines cholecystokinin secretion and effect on human gastric motility. Gastroenterology 116, 46–53 10.1016/S0016-5085(99)70227-1 9869601

[67] Feltrin K.L., Little T.J., Meyer J.H., Horowitz M., Smout A.J., Wishart J. et al. (2004) Effects of intraduodenal fatty acids on appetite, antropyloroduodenal motility, and plasma CCK and GLP-1 in humans vary with their chain length. Am. J. Physiol. Regul. Integr. Comp. Physiol. 287, R524–R533 10.1152/ajpregu.00039.2004 15166004

[68] Ross S.A. and Shaffer E.A. (1981) The importance of triglyceride hydrolysis for the release of gastric inhibitory polypeptide. Gastroenterology 80, 108–111 10.1016/0016-5085(81)90199-2 7450396

[69] Mandøe M.J., Hansen K.B., Hartmann B., Rehfeld J.F., Holst J.J. and Hansen H.S. (2015) The 2-monoacylglycerol moiety of dietary fat appears to be responsible for the fat-induced release of GLP-1 in humans. Am. J. Clin. Nutr. 102, 548–555 10.3945/ajcn.115.106799 26178726

[70] Thomsen C., Rasmussen O., Lousen T., Holst J.J., Fenselau S., Schrezenmeir J. et al. (1999) Differential effects of saturated and monounsaturated fatty acids on postprandial lipemia and incretin responses in healthy subjects. Am. J. Clin. Nutr. 69, 1135–1143 10.1093/ajcn/69.6.1135 10357731

[71] Kwasowski P., Flatt P.R., Bailey C.J. and Marks V. (1985) Effects of fatty acid chain length and saturation on gastric inhibitory polypeptide release in obese hyperglycaemic (ob/ob) mice. Biosci. Rep. 5, 701–705 10.1007/BF01117003 4063474

[72] Beysen C., Karpe F., Fielding B.A., Clark A., Levy J.C. and Frayn K.N. (2002) Interaction between specific fatty acids, GLP-1 and insulin secretion in humans. Diabetologia 45, 1533–1541 10.1007/s00125-002-0964-9 12436337

[73] Kuah R., Wang M.T., Yang Z., Back G., Li R.J.W., Bruce K. et al. (2025) Metformin boosts intestinal lipid sensing via GIP to suppress feeding. Diabetes 74, 1906–1917 10.2337/db25-0100 40694520

[74] Guccio N., Alcaino C., Miedzybrodzka E.L., Santos-Hernández M., Smith C.A., Davison A. et al. (2025) Molecular mechanisms underlying glucose-dependent insulinotropic polypeptide secretion in human duodenal organoids. Diabetologia 68, 217–230 10.1007/s00125-024-06293-3 39441374 PMC11663192

[75] Alcaino C., Guccio N., Miedzybrodzka E.L., Quale J.R., Lu T., Davison A. et al. (2025) Mechanisms of activation and serotonin release from human enterochromaffin cells. Cell Mol. Gastroenterol Hepatol. 19, 101610 10.1016/j.jcmgh.2025.101610 40812684 PMC12547937

[76] Reimann F., Diakogiannaki E., Hodge D. and Gribble F.M. (2020) Cellular mechanisms governing glucose-dependent insulinotropic polypeptide secretion. Peptides 125, 170206 10.1016/j.peptides.2019.170206 31756367

[77] Iwasaki K., Harada N., Sasaki K., Yamane S., Iida K., Suzuki K. et al. (2015) Free fatty acid receptor GPR120 is highly expressed in enteroendocrine K cells of the upper small intestine and has a critical role in GIP secretion after fat ingestion. Endocrinology 156, 837–846 10.1210/en.2014-1653 25535828

[78] Sankoda A., Harada N., Iwasaki K., Yamane S., Murata Y., Shibue K. et al. (2017) Long-chain free fatty acid receptor GPR120 mediates oil-induced gip secretion through CCK in male mice. Endocrinology 158, 1172–1180 10.1210/en.2017-00090 28324023

[79] Sankoda A., Harada N., Kato T., Ikeguchi E., Iwasaki K., Yamane S. et al. (2019) Free fatty acid receptors, G protein-coupled receptor 120 and G protein-coupled receptor 40, are essential for oil-induced gastric inhibitory polypeptide secretion. J. Diabetes Investig. 10, 1430–1437 10.1111/jdi.13059 31002464 PMC6825923

[80] Okawa M., Fujii K., Ohbuchi K., Okumoto M., Aragane K., Sato H. et al. (2009) Role of MGAT2 and DGAT1 in the release of gut peptides after triglyceride ingestion. Biochem. Biophys. Res. Commun. 390, 377–381 10.1016/j.bbrc.2009.08.167 19732742

[81] Chu Z.L., Carroll C., Alfonso J., Gutierrez V., He H., Lucman A. et al. (2008) A role for intestinal endocrine cell-expressed g protein-coupled receptor 119 in glycemic control by enhancing glucagon-like peptide-1 and glucose-dependent insulinotropic peptide release. Endocrinology 149, 2038–2047 10.1210/en.2007-0966 18202141

[82] Hodge D., Glass L.L., Diakogiannaki E., Pais R., Lenaghan C., Smith D.M. et al. (2016) Lipid derivatives activate GPR119 and trigger GLP-1 secretion in primary murine L-cells. Peptides 77, 16–20 10.1016/j.peptides.2015.06.012 26144594 PMC4788502

[83] Lan H., Vassileva G., Corona A., Liu L., Baker H., Golovko A. et al. (2009) GPR119 is required for physiological regulation of glucagon-like peptide-1 secretion but not for metabolic homeostasis. J. Endocrinol. 201, 219–230 10.1677/JOE-08-0453 19282326

[84] McGlone E.R., Malallah K., Cuenco J., Wewer Albrechtsen N.J., Holst J.J., Vincent R.P. et al. (2021) Differential effects of bile acids on the postprandial secretion of gut hormones: a randomized crossover study. Am. J. Physiol. Endocrinol. Metab. 320, E671–E679 10.1152/ajpendo.00580.2020 33459181

[85] Tough I.R., Schwartz T.W. and Cox H.M. (2020) Synthetic G protein-coupled bile acid receptor agonists and bile acids act via basolateral receptors in ileal and colonic mucosa. Neurogastroenterol. Motil. 32, e13943 10.1111/nmo.13943 32656959

[86] Brønden A., Albér A., Rohde U., Gasbjerg L.S., Rehfeld J.F., Holst J.J. et al. (2018) The bile acid-sequestering resin sevelamer eliminates the acute GLP-1 stimulatory effect of endogenously released bile acids in patients with type 2 diabetes. Diabetes Obes. Metab. 20, 362–369 10.1111/dom.13080 28786523

[87] Sommer C.A. and Mostoslavsky G. (2014) RNA-Seq analysis of enteroendocrine cells reveals a role for FABP5 in the control of GIP secretion. Mol. Endocrinol. 28, 1855–1865 10.1210/me.2014-1194 25268051 PMC5414789

[88] Han Y.E., Kang C.W., Oh J.H., Park S.H., Ku C.R., Cho Y.H. et al. (2018) Olfactory receptor OR51E1 mediates GLP-1 secretion in human and rodent enteroendocrine L cells. J. Endocr. Soc. 2, 1251–1258 10.1210/js.2018-00165 30402589 PMC6215084

[89] Goldspink D.A., Lu V., Miedzybrodzka E.L., Smith C.A., Foreman R.E., Billing L.J. et al. (2020) Labeling and characterization of human GLP-1-secreting L-cells in primary ileal organoid culture. Cell Reports 31, 107833 10.1016/j.celrep.2020.107833 32610134 PMC7342002

[90] Hodge D., Marsh W.J., Parker H.E., Ogunnowo-Bada E., Riches C.H., Habib A.M. et al. (2012) Somatostatin receptor 5 and cannabinoid receptor 1 activation inhibit secretion of glucose-dependent insulinotropic polypeptide from intestinal K cells in rodents. Diabetologia 55, 3094–3103 10.1007/s00125-012-2663-5 22872212 PMC3464380

[91] Tazoe H., Otomo Y., Karaki S., Kato I., Fukami Y., Terasaki M. et al. (2009) Expression of short-chain fatty acid receptor GPR41 in the human colon. Biomed. Res. 30, 149–156 10.2220/biomedres.30.149 19574715

[92] Samuel B.S., Shaito A., Motoike T., Rey F.E., Backhed F., Manchester J.K. et al. (2008) Effects of the gut microbiota on host adiposity are modulated by the short-chain fatty-acid binding G protein-coupled receptor, Gpr41. Proc. Natl. Acad. Sci. U.S.A. 105, 16767–16772 10.1073/pnas.0808567105 18931303 PMC2569967

[93] Nishida A., Miyamoto J., Shimizu H. and Kimura I. (2021) Gut microbial short-chain fatty acids-mediated olfactory receptor 78 stimulation promotes anorexigenic gut hormone peptide YY secretion in mice. Biochem. Biophys. Res. Commun. 557, 48–54 10.1016/j.bbrc.2021.03.167 33862459

[94] Abaffy T., Fu O., Harume-Nagai M., Goldenberg J.M., Kenyon V. and Kenakin T. (2024) Intracellular allosteric antagonist of the olfactory receptor OR51E2. Mol. Pharmacol. 106, 21–32 10.1124/molpharm.123.000843 38719475 PMC11187688

[95] Wu C., Jeong M.Y., Kim J.Y., Lee G., Kim J.S., Cheong Y.E. et al. (2021) Activation of ectopic olfactory receptor 544 induces GLP-1 secretion and regulates gut inflammation. Gut Microbes 13, 1987782 10.1080/19490976.2021.1987782 34674602 PMC8632334

[96] Tanaka T., Katsuma S., Adachi T., Koshimizu T.A., Hirasawa A. and Tsujimoto G. (2008) Free fatty acids induce cholecystokinin secretion through GPR120. Naunyn Schmiedebergs Arch. Pharmacol. 377, 523–527 10.1007/s00210-007-0200-8 17972064

[97] Liou A.P., Lu X.P., Sei Y., Zhao X.L., Pechhold S., Carrero R.J. et al. (2011) The G-protein-coupled receptor GPR40 directly mediates long-chain fatty acid-induced secretion of cholecystokinin. Gastroenterology 140, 903–912 10.1053/j.gastro.2010.10.012 20955703 PMC4717904

[98] Mandoe M.J., Hansen K.B., Windelov J.A., Knop F.K., Rehfeld J.F., Rosenkilde M.M. et al. (2018) Comparing olive oil and C4-dietary oil, a prodrug for the GPR119 agonist, 2-oleoyl glycerol, less energy intake of the latter is needed to stimulate incretin hormone secretion in overweight subjects with type 2 diabetes. Nutr. Diab 8, 2 10.1038/s41387-017-0011-z PMC6199285 29330461

[99] Ochiai K., Hirooka R., Sakaino M., Takeuchi S. and Hira T. (2021) 2-Arachidonoyl glycerol potently induces cholecystokinin secretion in murine enteroendocrine STC-1 cells via cannabinoid receptor CB1. Lipids 56, 603–611 10.1002/lipd.12323 34533218

[100] Foltin R.W., Brady J.V. and Fischman M.W. (1986) Behavioral analysis of marijuana effects on food intake in humans. Pharmacol. Biochem. Behav. 25, 577–582 10.1016/0091-3057(86)90144-9 3774823

[101] Van Gaal L.F., Rissanen A.M., Scheen A.J., Ziegler O. and Rössner S. (2005) Effects of the cannabinoid-1 receptor blocker rimonabant on weight reduction and cardiovascular risk factors in overweight patients: 1-year experience from the RIO-Europe study. Lancet 365, 1389–1397 10.1016/S0140-6736(05)66374-X 15836887

[102] Kaelberer M.M., Buchanan K.L., Klein M.E., Barth B.B., Montoya M.M., Shen X. et al. (2018) A gut-brain neural circuit for nutrient sensory transduction. Science 361, eaat5236 10.1126/science.aat5236 30237325 PMC6417812

[103] Buchanan K.L., Rupprecht L.E., Kaelberer M.M., Sahasrabudhe A., Klein M.E., Villalobos J.A. et al. (2022) The preference for sugar over sweetener depends on a gut sensor cell. Nat. Neurosci. 25, 191–200 10.1038/s41593-021-00982-7 35027761 PMC8825280

[104] Li M., Tan H.-E., Lu Z., Tsang K.S., Chung A.J. and Zuker C.S. (2022) Gut–brain circuits for fat preference. Nature 610, 722–730 10.1038/s41586-022-05266-z 36070796 PMC9605869

[105] Thomas A.S., Sassi M., Angelini R., Morgan A.H. and Davies J.S. (2022) Acylation, a conductor of ghrelin function in brain health and disease. Front Physiol. 13, 831641 10.3389/fphys.2022.831641 35845996 PMC9280358

[106] Nishi Y., Hiejima H., Hosoda H., Kaiya H., Mori K., Fukue Y. et al. (2005) Ingested medium-chain fatty acids are directly utilized for the acyl modification of ghrelin. Endocrinology 146, 2255–2264 10.1210/en.2004-0695 15677766

[107] Yamato M., Sakata I., Wada R., Kaiya H. and Sakai T. (2005) Exogenous administration of octanoic acid accelerates octanoylated ghrelin production in the proventriculus of neonatal chicks. Biochem. Biophys. Res. Commun. 333, 583–589 10.1016/j.bbrc.2005.05.107 15953586

[108] Bando M., Iwakura H., Koyama H., Hosoda H., Shigematsu Y., Ariyasu H. et al. (2016) High incorporation of long-chain fatty acids contributes to the efficient production of acylated ghrelin in ghrelin-producing cells. FEBS Lett. 590, 992–1001 10.1002/1873-3468.12132 26991015

[109] Ikenoya C., Takemi S., Kaminoda A., Aizawa S., Ojima S., Gong Z. et al. (2018) β-Oxidation in ghrelin-producing cells is important for ghrelin acyl-modification. Sci. Rep. 8, 9176 10.1038/s41598-018-27458-2 29907775 PMC6003948

[110] Janssen S., Laermans J., Iwakura H., Tack J. and Depoortere I. (2012) Sensing of fatty acids for octanoylation of ghrelin involves a gustatory G-protein. PloS ONE 7, e40168 10.1371/journal.pone.0040168 22768248 PMC3387020

[111] Kirchner H., Gutierrez J.A., Solenberg P.J., Pfluger P.T., Czyzyk T.A., Willency J.A. et al. (2009) GOAT links dietary lipids with the endocrine control of energy balance. Nat. Med. 15, 741–745 10.1038/nm.1997 19503064 PMC2789701

[112] Janssen S., Laermans J., Verhulst P.J., Thijs T., Tack J. and Depoortere I. (2011) Bitter taste receptors and α-gustducin regulate the secretion of ghrelin with functional effects on food intake and gastric emptying. Proc. Natl. Acad. Sci. U.S.A. 108, 2094–2099 10.1073/pnas.1011508108 21245306 PMC3033292

[113] Janssen S. and Depoortere I. (2013) Nutrient sensing in the gut: new roads to therapeutics? Trends Endocrinol. Metab. 24, 92–100 10.1016/j.tem.2012.11.006 23266105

[114] Monteleone P., Bencivenga R., Longobardi N., Serritella C. and Maj M. (2003) Differential responses of circulating ghrelin to high-fat or high-carbohydrate meal in healthy women. J. Clin. Endocrinol. Metab. 88, 5510–5514 10.1210/jc.2003-030797 14602798

[115] Foster-Schubert K.E., Overduin J., Prudom C.E., Liu J., Callahan H.S., Gaylinn B.D. et al. (2008) Acyl and total ghrelin are suppressed strongly by ingested proteins, weakly by lipids, and biphasically by carbohydrates. J. Clin. Endocrinol. Metab. 93, 1971–1979 10.1210/jc.2007-2289 18198223 PMC2386677

[116] Overduin J., Frayo R.S., Grill H.J., Kaplan J.M. and Cummings D.E. (2005) Role of the duodenum and macronutrient type in ghrelin regulation. Endocrinology 146, 845–850 10.1210/en.2004-0609 15528308

[117] Lund M.L., Egerod K.L., Engelstoft M.S., Dmytriyeva O., Theodorsson E., Patel B.A. et al. (2018) Enterochromaffin 5-HT cells—a major target for GLP-1 and gut microbial metabolites. Mol. Metab. 11, 70–83 10.1016/j.molmet.2018.03.004 29576437 PMC6001397

[118] Yano J.M., Yu K., Donaldson G.P., Shastri G.G., Ann P., Ma L. et al. (2015) Indigenous bacteria from the gut microbiota regulate host serotonin biosynthesis. Cell 161, 264–276 10.1016/j.cell.2015.02.047 25860609 PMC4393509

[119] Reigstad C.S., Salmonson C.E., Rainey J.F., Szurszewski J.H., Linden D.R., Sonnenburg J.L. et al. (2015) Gut microbes promote colonic serotonin production through an effect of short-chain fatty acids on enterochromaffin cells. FASEB J. 29, 1395–1403 10.1096/fj.14-259598 25550456 PMC4396604

[120] Alemi F., Poole D.P., Chiu J., Schoonjans K., Cattaruzza F., Grider J.R. et al. (2013) The receptor TGR5 mediates the prokinetic actions of intestinal bile acids and is required for normal defecation in mice. Gastroenterology 144, 145–154 10.1053/j.gastro.2012.09.055 23041323 PMC6054127

[121] Dakal T.C., Xiao F., Bhusal C.K., Sabapathy P.C., Segal R., Chen J. et al. (2025) Lipids dysregulation in diseases: core concepts, targets and treatment strategies. Lipids Health Dis. 24, 61 10.1186/s12944-024-02425-1 39984909 PMC11843775

[122] Nauck M.A. and Meier J.J. (2018) Incretin hormones: their role in health and disease. Diabetes Obes. Metab. 20, 5–21 10.1111/dom.13129 29364588

[123] Higgins V. and Adeli K. (2017) Postprandial dyslipidemia: pathophysiology and cardiovascular disease risk assessment. EJIFCC 28, 168–184 29075168 PMC5655632

[124] Marso S.P., Bain S.C., Consoli A., Eliaschewitz F.G., Jódar E., Leiter L.A. et al. (2016) Semaglutide and cardiovascular outcomes in patients with type 2 diabetes. N. Engl. J. Med. 375, 1834–1844 10.1056/NEJMoa1607141 27633186

[125] Marso S.P., Daniels G.H., Brown-Frandsen K., Kristensen P., Mann J.F., Nauck M.A. et al. (2016) Liraglutide and cardiovascular outcomes in type 2 diabetes. N. Engl. J. Med. 375, 311–322 10.1056/NEJMoa1603827 27295427 PMC4985288

[126] Xiao C., Bandsma R.H., Dash S., Szeto L. and Lewis G.F. (2012) Exenatide, a glucagon-like peptide-1 receptor agonist, acutely inhibits intestinal lipoprotein production in healthy humans. Arterioscler. Thromb. Vasc. Biol. 32, 1513–1519 10.1161/ATVBAHA.112.246207 22492091

[127] Xiao C., Dash S., Morgantini C., Patterson B.W. and Lewis G.F. (2014) Sitagliptin, a DPP-4 inhibitor, acutely inhibits intestinal lipoprotein particle secretion in healthy humans. Diabetes 63, 2394–2401 10.2337/db13-1654 24584549 PMC4066342

[128] Tremblay A.J., Lamarche B., Deacon C.F., Weisnagel S.J. and Couture P. (2011) Effect of sitagliptin therapy on postprandial lipoprotein levels in patients with type 2 diabetes. Diabetes Obes. Metab. 13, 366–373 10.1111/j.1463-1326.2011.01362.x 21226820

[129] Dahl K., Brooks A., Almazedi F., Hoff S.T., Boschini C. and Baekdal T.A. (2021) Oral semaglutide improves postprandial glucose and lipid metabolism, and delays gastric emptying, in subjects with type 2 diabetes. Diab. Obes. Metab. 23, 1594–1603 10.1111/dom.14373 PMC8251575 33710717

[130] Bu T., Sun Z., Pan Y., Deng X. and Yuan G. (2024) Glucagon-like peptide-1: new regulator in lipid metabolism. Diab. Metab. J. 48, 354–372 10.4093/dmj.2023.0277 PMC11140404 38650100

[131] Sebhat I.K., Murphy M.J.M., Zheng S., Lovelett R.J., Engelstoft M., Kosinski D. et al. (2026) Gut enteroendocrine cell activation using a combination of GPR119 and GPR40 agonists results in synergistic hormone secretion in mice and humans. Cell Metab. 38, 50–64.e12 10.1016/j.cmet.2025.11.001 41338185

[132] Crutchlow M., Liu J., Hernandez-Illas M., Zhang H., Arreglado A., Vance A. et al. (2025) Combined GPR40 and GPR119 full agonism with K-757 and K-833 results in robust glucose lowering and modest weight loss in overweight/obese subjects with T2DM. Diab. Obes. Metab. 27, 6264–6274 10.1111/dom.70014 40767101

[133] Crutchlow M., Liu J., Romero C., Watkins E., Zhang H., Arreglado A. et al. (2025) Combined agonism of nutrient receptors GPR40 and GPR119 with K-757 and K-833 results in weight loss and blood pressure reductions in obese subjects without type 2 diabetes mellitus. Diab. Obes. Metab. 27, 7198–7209 10.1111/dom.70120 40954563

[134] Heck A.M., Yanovski J.A. and Calis K.A. (2000) Orlistat, a new lipase inhibitor for the management of obesity. Pharmacotherapy 20, 270–279 10.1592/phco.20.4.270.34882 10730683 PMC6145169

[135] Thomas C., Gioiello A., Noriega L., Strehle A., Oury J., Rizzo G. et al. (2009) TGR5-mediated bile acid sensing controls glucose homeostasis. Cell Metab. 10, 167–177 10.1016/j.cmet.2009.08.001 19723493 PMC2739652

[136] Chiang J.Y. (2013) Bile acid metabolism and signaling. Compr. Physiol. 3, 1191–1212 10.1002/j.2040-4603.2013.tb00517.x 23897684 PMC4422175

[137] Chiang J.Y.L. and Ferrell J.M. (2020) Bile acid receptors FXR and TGR5 signaling in fatty liver diseases and therapy. Am. J. Physiol. Gastrointest. Liver Physiol. 318, G554–G573 10.1152/ajpgi.00223.2019 31984784 PMC7099488

[138] Larraufie P., Roberts G.P., McGavigan A.K., Kay R.G., Li J., Leiter A. et al. (2019) Important role of the GLP-1 axis for glucose homeostasis after bariatric surgery. Cell Rep. 26, 1399–1408.e6 10.1016/j.celrep.2019.01.047 30726726 PMC6367566

[139] Cai M., Tejpal S., Tashkova M., Ryden P., Perez-Moral N., Saha S. et al. (2025) Upper-gastrointestinal tract metabolite profile regulates glycaemic and satiety responses to meals with contrasting structure: a pilot study. Nat. Metab. 7, 1459–1475 10.1038/s42255-025-01309-7 40542296 PMC12286859

[140] Holst J.J. and Madsbad S. (2016) Mechanisms of surgical control of type 2 diabetes: GLP-1 is key factor. Surg. Obes. Relat. Dis. 12, 1236–1242 10.1016/j.soard.2016.02.033 27313194

[141] Akalestou E., Miras A.D., Rutter G.A. and le Roux C.W. (2022) Mechanisms of weight loss after obesity surgery. Endocr. Rev. 43, 19–34 10.1210/endrev/bnab022 34363458 PMC8755990

[142] Chao J., Coleman R.A., Keating D.J. and Martin A.M. (2025) Gut microbiome regulation of gut hormone secretion. Endocrinology 166, bqaf004 10.1210/endocr/bqaf004 40037297 PMC11879239

